# Immunological evaluation of *Lactobacillus casei *Zhang: a newly isolated strain from koumiss in Inner Mongolia, China

**DOI:** 10.1186/1471-2172-9-68

**Published:** 2008-11-19

**Authors:** Tuo Ya, Qijin Zhang, Fuliang Chu, Justin Merritt, Menhe Bilige, Tiansong Sun, Ruiting Du, Heping Zhang

**Affiliations:** 1 The Key Laboratory of Dairy Biotechnology and Bioengineering, Education Ministry of PR China, Department of Food Science and Engineering, Inner Mongolia Agricultural University, Huhhot 010018, PR China; 2 Clinical Testing Center, Inner Mongolia Medical College affiliated hospital, Huhhot 010050, PR China; 3 Institute of Microbiology, Chinese Academy of Sciences, Beijing 100101, PR China; 4 College of Dentistry, University of Oklahoma Health Sciences Centre, 975 NE 10th St BRC364, Oklahoma City, OK 73104-5419, USA

## Abstract

**Background:**

There is increasing evidence to suggest an immunomodulation function both within the intestines and systemically upon consuming probiotic species. We recently isolated a novel LAB, *Lactobacillus casei*Zhang (LcZhang) from koumiss. LcZhang exhibited favorable probiotic properties, such as acid resistance, bile resistance, gastrointestinal (GI) colonization ability, etc. In order to examine the immunomodulatory qualities of LcZhang, we administered LcZhang to healthy mice with varying doses of either live or heat-killed LcZhang and measured various parameters of the host immune response.

**Results:**

The study was performed in four separate experiments via oral administration of live and heat-killed LcZhang to BALB/c mice for several consecutive days. We investigated the immunomodulating capacity of LcZhang *in vivo *by analyzing the profile of cytokines, T cell subpopulations, and immunoglobulin concentrations induced in blood serum and intestinal fluid in BALB/c mice. Only live bacteria elicited a wide range of immune responses, which include the increased production of interferon-γ (IFN-γ), and depression of tumor necrosis factor-α (TNF-α) levels. In addition, interleukin-2 (IL-2) and IL-2 receptor gene transcription increased significantly, but the proportion of T cell subsets appeared to be unaffected. We also observed that LcZhang was capable of inducing gut mucosal responses by enhancing the production of secretory Immunoglobulin A (sIgA) as well influencing the systemic immunity via the cytokines released to the circulating blood.

**Conclusion:**

The present work shows that the dose-dependent administration of LcZhang is capable of influencing immune responses, implying that it may be a valuable strain for probiotic use in humans.

## Background

The association between improved health and probiotic bacteria consumption was first documented more than a century ago. Since then, numerous species of bacteria have been examined for their potential health benefits, particularly within the late two decades [1,2]. Data from these studies suggest that the effects upon the host might be attributable to the probiotic bacteria themselves through an immunomodulatory activity. These species are able to survive in the human gastrointestinal (GI) tract and influence the host enteric microbiota, which subsequently modulate host immunophysiologic responses. In addition, numerous *in vitro*, animal model, and human studies also suggest that many of these same species are capable of producing a significant impact upon the host immune system as well [1,2].

As described by Vinderola *et al*., the two primary routes that probiotic species are able to impact the host are through their direct colonization of the host as well as indirectly by the subsequent release of various metabolites from these species [3]. The former functions via an influence upon the balance of the gut microbial environment. Whereas, the latter route functions through more complex pathways, such as immune recognition of bacterial envelope components, activation of Toll-like receptors, CD14, mannose receptors, etc. [4]. These events have been demonstrated to promote the rapid activation of the NF-κB and STAT transduction pathways, which subsequently induces the production of pro-inflammatory cytokines [5]. In fact, many characterized probiotic effects are mediated through immune regulation, particularly through perturbations in the balance of pro-inflammatory and anti-inflammatory cytokines [6]. Thus, probiotics can be employed therapeutically to alleviate intestinal inflammation, normalize gut mucosal dysfunction, and down-regulate hypersensitivity reactions [6]. Furthermore, this approach also has a robust safety record. Thus, probiotics have many properties that make them highly desirable for the prevention and/or treatment of a wide range of ailments [7].

Previous data has suggested that careful attention should be utilized when selecting strains to be used for probiotic therapy, as differences do exist in the immunomodulatory capacity of bacterial strains. Consequently, we were particularly interested in isolating a probiotic strain with both strong probiotic potential as well as immunomodulatory activity. To this end, we recently isolated a novel LAB, *Lactobacillus casei *Zhang (LcZhang) from koumiss (a kind of homemade fermented horse milk beverage) widely used in traditional Mongolian medicine in the Inner Mongolia region of China [8-10]. LcZhang exhibited favorable probiotic properties, such as acid resistance, bile resistance, GI colonization ability, etc. [11]. In order to elucidate the immunomodulatory effects of LcZhang, we administered healthy mice with varying doses of either live or heat-killed LcZhang and measured the level of various cytokines, immunoglobulin G (IgG), and T-cell populations in the serum, IL-2 and IL-2 receptor gene expression in the spleen, and secretory immunoglobulin A (sIgA) in the intestine.

## Results

### Live LcZhang elicits IFN-γ production and inhibits TNF-α production in a dose-dependent fashion

**Figure 1:**
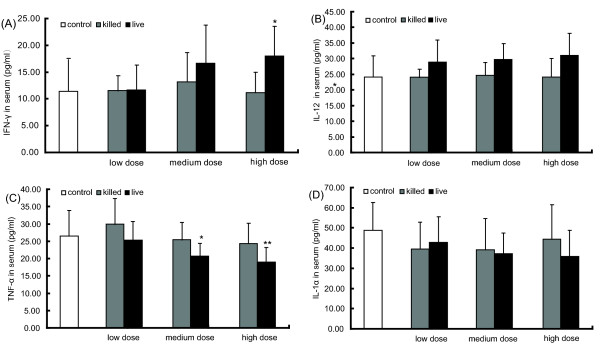
**Effects of the oral administration of varying doses of LcZhang on cytokines secretion**. Induction of major immunoregulatory cytokines (A: IFN-γ; B: IL-12; C: TNF-α; D: IL-1α) levels in serum by live LcZhang and heat-killed bacteria after 15 days oral administration. Data represent the mean ± standard deviation of each cytokine in pg/ml in serum for each test treatment (*n *= 10). Three dosages of bacteria were shown: low dose means 0.25 × 10^9 ^cells/ml; medium dose means 0.5 × 10^9 ^cells/ml; high dose means 1.0 × 10^9 ^cells/ml. "Control" refers to the same volume of PBS (open column); "killed" represents heat killed LcZhang (grey column); "live" represents live LcZhang (solid column). Data are means ± S.D. Error bars represent S.D. *P < 0.05; **P < 0.01.

*Lcactobacillus *Zhang is initially isolated strain from homemade koumiss and determined to be *Lactobacillus casei *using 16 S rRNA-based phylogenetic analysis together with a wide array of biochemical assays [8]. In order to examine the immunomodulating capacity of LcZhang, we began by examining its effect upon cytokine levels in the circulating blood of healthy mice. We quantified IFN-γ and IL-12 levels in the sera of mice receiving a daily dose of LcZhang for 15 consecutive days. The level of IFN-γ increased in all the three dose groups (Fig. 1A). However, only the high dose group elicited a significantly higher response (P < 0.05) compared to the heat killed group and control group (Fig. 1A). IL-12 also increased, but none of groups exhibited a significant difference (Fig. 1B).

As shown in figure 1C, TNF-α production was significantly reduced with a high dose (P < 0.01) and medium dose of LcZhang (P < 0.05). The effect of LcZhang administration on IL-1α production is shown in figure 1D. No significant changes were observed, though IL-1α levels were slightly reduced. In addition, the effects were dose-dependent. No significant changes were observed to heat killed LcZhang administration (Fig. 1).

### IL-2 and IL-2 receptor gene transcription

**Figure 2:**
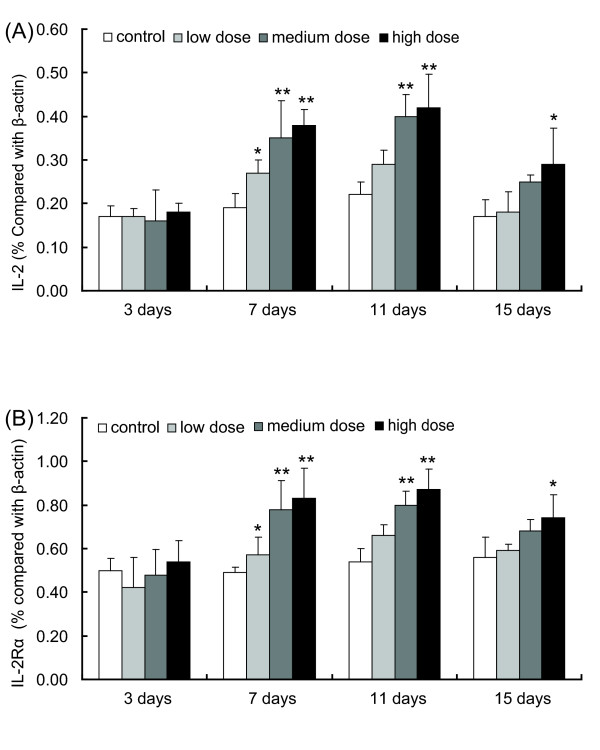
**Analysis of the IL-2 and IL-2R mRNA level in spleen by reverse transcription-PCR**. Semi-quantitative detection of IL-2 and IL-2 receptor gene transcription before and after oral administration of live LcZhang with three dosages compared to the gene transcription of β-actin. A: IL-2 gene transcription; B: IL-2 receptor gene transcription. The relative levels of IL-2 and IL-2R mRNA in mouse spleen was calculated and expressed at the percentage of mRNA to β-actin. The control (open column) used normal PEF food with 1.0 ml PBS. Three dosages of bacteria were shown: a low dose means 1 ml × 0.25 × 10^9 ^cells/ml (grey column); a medium dose was 1 ml × 0.5 × 10^9 ^cells/ml (dark grey column); a high dose was 1 ml × 1.0 × 10^9 ^cells/ml (solid column). Data are means ± S.D. (*n *= 5). Error bars represent S.D. *P < 0.05; **P < 0.01.

In addition to circulating cytokine levels, we were also interested to determine whether LcZhang had the ability to affect cytokine transcription as well. Therefore, IL-2 and IL-2 receptor gene transcription were measured after the intake of LcZhang. As shown in figure 2A, significant increases in IL-2 gene transcription were detected at day 7 and 11 with all dose groups and at day 11, IL-2 mRNA levels peaked at almost double the control. By day 15, both IL-2 and IL-2 receptor gene transcription had decreased with only the high dose samples remaining significantly different from the control (Fig. 2A and 2B). Throughout the assay period, IL-2R gene transcription showed a similar trend with that of IL-2 (Fig. 2B).

### T cell subpopulation patterns

The proportion of CD3+, CD4+, and CD8+ T cells tended to increase slightly over the assay period, but the differences were not significant. Both CD4+, and CD8+ T-cells and the ratio of CD4+/CD8+ did not seem to be affected by LcZhang (Data not shown).

### Intestinal sIgA production

**Figure 3:**
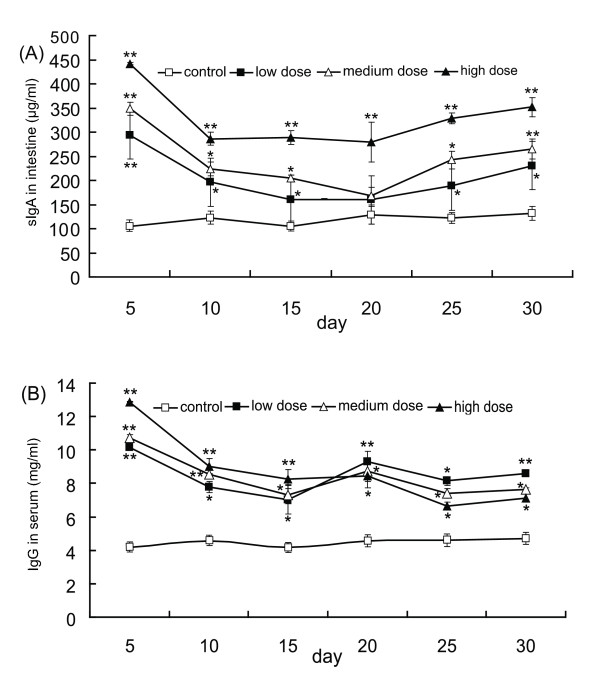
**Time course of the effects of oral administration of varying dosages of LcZhang on immunoglobulin production in serum and in the intestines**. ELISA detected sIgA level in intestine content and IgG level in serum at every 5 days after oral administration of live LcZhang. A: sIgA levels in intestine content; B: IgG levels in serum. Control refers 2.0 ml PBS (open squares); low dose represents 2.0 ml live cells with density of 0.5 × 10^9 ^cells/ml (solid squares); medium dose represents 2.0 ml live cells with density of 1 × 10^9 ^cells/ml (open triangles); high dose represents 2.0 ml live cells with density of 2 × 10^9 ^cells/ml (solid triangles). Five mice were sacrificed in every 5 days. Data are means ± S.D. (*n *= 5). Error bars represent S.D. *P < 0.05; **P < 0.01.

Intestinal content from the caecum and distal colonic sections were collected from each mouse and the sIgA concentration was measured (Fig. 3A). A significant increase in the concentration of this immunoglobulin was also observed at all dose groups compared to the control. Similar to that of IgG, by the fifth day, sIgA levels peaked and subsequently decreased to a relatively stable level. However, from days 20 – 30, a slight increase in the level of sIgA was observed for each of the dose groups (Fig. 3A). At its peak level on day 5, the high dose group exhibited 4-fold increase in sIgA over the control, while medium and low dose groups both showed about a 3-fold increase.

### IgG production in serum

Figure 3B shows significantly increased IgG (P < 0.01) at every dose group in the serum after ingesting LcZhang. By the fifth day after LcZhang ingestion, IgG levels had also peaked for each dose group with the high dose group exhibiting a 3-fold increase over the control. This level decreased to about 8 mg/ml for each dose group after day 10 and remained relatively constant for the remainder of the assay period. Interestingly, the IgG level of the high dose was slightly lower than the medium and low dose groups after the twentieth day (Fig. 3B).

## Discussion

The potential health benefits of consuming lactic acid bacteria (LAB) are increasingly being recognized. In fact, there are some suggestions that LAB commonly used in the dairy industry are therapeutic for diseases such as cancer, various infections, GI disorders, asthma, etc. Because the immune system is an important contributor to each of these diseases, the immunoregulatory effects of LAB were of primary interest. In those studies, cytokine production, antibody (sIgA, IgG, and IgE) production, phagocytic activity, Peyer's patch (PP) activation, T cell function, and natural killer (NK) cell activity were shown to increase with LAB consumption or when cells were exposed to LAB *in vitro *or *in vivo *[6]. It is noted that probiotics interferes basal lymphoproliferation or mitogen-stimulated T- and B-cell proliferation [12,13].

The present study focuses on the immunoregulating capacity of LcZhang, which is a nice probiotic candidate [8]. Our data showed that the pro-inflammatory cytokines IFN-γ can be increased in a dose dependent manner after the intake of viable LcZhang. IFN-γ is a typical type 1 helper T cell (Th1) cytokine and the augmentation of Th1 cytokines are known to enhance various immune responses. IL-12 stimulates the production of IFN-γ and is at least partially associated with the production of Th1 cells. Furthermore, probiotics have been suggested to affect DC maturation and functioning [14]. LABs may potentially influence the development of Th1 and Th2 via changes in DC types and/or the extent of maturation [15]. Usually, the immunoregulatory effects of probiotics can be ascribed to a skew of the Th1/Th2 balance in favor of Th1 mediated immunity [4,16,17]. Consequently, the release of the Th1 associated cytokines IL-12, IFN-γ and TNF-α by various cell types has been demonstrated to be increased by LAB [18], which could help to mediate cellular immunity and inhibit the proliferation of Th2 [19,20]. Unexpectedly, our results with LcZhang do not completely agree with those reported previously. Specifically, a reduction of TNF-α was observed in the serum. As a pro-inflammatory cytokine, TNF-α plays a pivotal role in inflammation, mediating fevers, and inducing the liver to produce various detrimental proteins. In certain immune deficiency disorders, elevated TNF-α production has been demonstrated to be a mediator of some of the disease pathology [21]. Thus, LcZhang administration may have some potential utility to help moderate the inflammation associated with elevated TNF-α.

Also, via RT-PCR we detected a significant increase of IL-2 and IL-2 receptor gene transcription level after 7 days of oral administration of LcZhang. IL-2 can induce the proliferation of activated T- and B-cells, enhance NK cytotoxicity, and increase the killing activity of monocytes and macrophages for tumour cells and bacteria [13,20,22]. Increased IL-2 and IL-2 receptor transcription also implicates the activation of T cells. Collectively, oral administration of LcZhang elicits a modest level of systemic immunoenhancing effects. In this study, the level of sIgA in the intestine was increased by the oral administration of LcZhang. Intestinal secretory IgA plays a principal role in the intestinal immune system: it prevents infection by inhibiting the attachment of bacteria and viruses to the gastrointestinal tract [23,24]. Since LcZhang was able to stably increase the level of sIgA for at least 30 days, it is conceivable that LcZhang may have the ability to improve the gastrointestinal mucosal resistance to infections [25]. Indeed, similar results have ever been found with other probiotic strains [23,24,26], but further studies are needed to verify whether this is also the case with LcZhang.

The glycoprotein immunoglobulin G (IgG) accounts for about 75% of the total immunoglobulins in plasma of healthy individuals. Immunoglobulin G (IgG) antibodies are predominately involved in the secondary antibody response, which occurs later following antigen recognition. The presence of specific IgG generally corresponds to maturation of antibody response. It is interesting that total IgG level in sera increased after administered LcZhang in the present study. With temporal increased IgG level, IgG level restored to normal level after 10 days administration of LcZhang, which is still significantly higher than control group. It is conceivable that some biological activate peptides released from LcZhang may involve in triggering secondary antibodies responses. The mechanism of action is still unknown, further investigation required to evaluate changes of IgG sub-classes, other classes of Ig, such as IgE or IgM, etc.

## Conclusion

In summary, this data indicated that oral administration of LcZhang is able to modulate immune responses. The effects exhibit strong dose-dependent characteristics and is consistently related to the viability of this bacteria. Also, oral administration of LcZhang effected the composition of the intestinal microbiota, which suggests a mechanism by which LcZhang could enhance specifically local as well as systemic immune responses. Further studies are necessary to better assess the full breadth of the immunomodulatory activities of LcZhang. In addition, it is of great interest to measure the effects of koumiss ingestion upon immune system activation in the local population of Inner Mongolia.

## Methods

### Bacteria strains and growth condition

*Lactobacillus casei *strain Zhang was maintained by subculturing in Trypticase Peptone Yeast broth (TPY; DIFCO, PQ, Canada) using 1% (vol/vol) inoculums and 24 h of incubation at 37°C and were stored at 4°C between transfers. Each culture was subcultured twice in the TPY broth before use [27].

Serial dilutions of freshly-prepared culture were plated onto Man Rogosa Sharpe (MRS) agar/cysteine-HCl and cultured for 36 h under anaerobic conditions, prior to enumeration. For all experiments involving killed bacterial preparations, samples of freshly-prepared cultures were enumerated using the appropriate agar, while additional samples of the same cultures were heat-killed (70°C/30 min). Subsequently, the concentration of the heat-killed preparation was adjusted in lieu of the live plate counts and used for further experimentation. Successful heat-killing was confirmed by the absence of bacterial growth on appropriate agar plates.

### Animal and treatment

Female Balb/c mice (6 weeks of age) with a body weight at about 18–22 g were purchased from the Center for Experimental Animals at the Agriculture University of Inner Mongolia. Guidelines for the care and use of animals were followed and approved by the Ethical Committee of the Agriculture University of Inner Mongolia. Five mice were kept in each cage. Each experimental group consisted of 10 mice in cytokines detection assays, 20 mice in IL-2 gene transcription assay while each experimental group consisted of 30 mice in T cell subpopulation detection and immunoglobulin concentration detection experiments. At each period, 5 mice were sacrificed. The animals were bred specific pathogen free (SPF) and kept in a temperature- and light-controlled environment. The animals were allowed free access to a non-purified MF diet and water until the experiment began. For cytokine measurements and gene transcription experiments, the bacterial suspensions were adjusted with PBS such that a low dose was 0.25 × 10^9 ^cells/ml; medium dose 0.5 × 10^9 ^cells/ml; high dose 1.0 × 10^9 ^cells/ml. Oral gavage volume was 1 ml per day. For assays of T cell subpopulations and immunoglobulin production, a low dose was adjusted to 0.50 × 10^9 ^cells/ml; a medium dose 1.0 × 10^9 ^cells/ml; and a high dose 2.0 × 10^9 ^cells/ml. Oral gavage volume was 2 ml per day. Every five days, 5 mice were sacrificed for immunoglobulin production experiments. Sera and intestinal contents were collected immediately.

### Detection of cytokine levels in sera

Blood was collected from the orbital cavity under diethyl ether anesthesia; sera were separated by centrifugation at 1500 rpm for 10 min at room temperature. Serum was stored at -70°C until further analyzed. The levels of cytokines in sera were determined by capture ELISAs and measured in each case against a standard curve generated by employing known amounts of recombinant cytokine. IFN-γ, IL-12, TNF-α and IL-1α were measured using ELISA detection kits (Senxiong Biotech Inc, Shanghai, China) according to the manufacturer's protocol. In all cases, detection antibody binding was visualized using streptavidin-horse radish peroxidase conjugate and an OPD (o-Phenylenediamine) substrate system at an OD of 492 nm. For each experiment, the levels of cytokine detected in serum by exposure to LcZhang were determined by comparing against the cytokine levels observed in the standard curves (controls). Data were calculated as the mean cytokine response (in 5 pg/ml) of each treatment from triplicate wells, plus or minus the standard deviation.

### Analysis of IL-2 and IL-2R gene transcription

Total RNA was isolated from the spleen with Trizol reagent (Invitrogen, USA) according to the manufacturer's instructions. Initially, a portion of the spleen was frozen in liquid nitrogen, placed in lysis buffer, and immediately disrupted and homogenized using a rotor-stator homogenizer. About 50–100 mg of spleen material was used for RNA isolation with 1 ml of Trizol Reagent. AMV reverse transcriptase (Promega, USA), and oligo-(dT) 15 primers (Promega) were used for generating total cDNA. mRNA levels were also measured by semi-quantitative reverse transcription-PCR. RNA concentration was assessed spectrophotometrically and RNA integrity was visualized with gel electrophoresis. The concentration of all mouse RNA samples was adjusted to 2.5 μg/μl by vacuum drying and resuspending in RNase free water (Takara, Japan). All RNA samples were stored at -80°C until used. One step RT-PCR was performed using the commercially available kit (High Fidelity RNA PCR Kit, Takara, Japan). IL-2 and IL-2R gene transcription were evaluated by semi-quantitative RT-PCR. The forward primer for IL-2 is 5'-CTGGAGCAGCTGTTGATG-3' and 5'-CGAATTGGCACTCAAATG-3'for the reverse. The size of the amplified IL-2 fragment was 286 base pair (bp). For the IL-2 receptor, the primers are 5'-CGGTTTCCGAAGACTAAA-3'and 5'-GTCCTTCCACGAAATGAT-3' for the reverse. The size of the amplified IL-2R fragment was 266 bp. β-actin gene transcription was used as a housekeeping control for normalization. The β-actin primer sequences are 5'-GTTACCAACTGGGACGACA-3' and 5'-AGGCATACAGGGACAGCA-3'. The size of the amplified β-actin fragment was 208 bp.

PCR was performed in a reaction volume of 50 μl, using the High Fidelity RNA PCR Kit (Takara, Japan) according to the instructions of the manufacturer. For PCR, the initial melting temperature was 94°C for 4 min, followed by 31 cycles at 94°C for 30 s, 55°C for 30 s and 72°C for 1 min, with a final extension at 72°C for 10 min. The PCR products were visualized on a 2% agarose gel. Amplicon band intensity was calculated using Image Analysis Software (Alpha Innotech Corporation).

### Measurement of Immunoglobulin concentration

Concentration of IgG in sera and sIgA from intestines were determined by ELISA quantitative kits (Sun biomedical Technology Beijng Co., Ltd, Beijing, China) according to manufacturer instructions. Immunoglobulin concentrations were evaluated every five days for a total of 30 days after the oral administration of LcZhang.

### Measurement of intestinal sIgA

Immediately after sacrificing the mice, samples from the caecum and distal sections of the intestine from each mouse were collected and homogenized in saline solution (50 mg/ml). Particulate material was removed by centrifugation at 10,000 ×g for 10 min at 4°C. The supernatant fluid was stored at -80°C for IgA measurement. The level of sIgA in supernatants were determined by ELISA quantitation kits (Sun biomedical Technology Co., Ltd, Beijng, China) according to manufacturer instructions.

### Measurement of sera IgG

Blood was collected from the orbital cavity under diethyl ether anesthesia and the sera were separated by centrifugation at 1500 rpm for 10 min at room temperature. The IgG concentration was determined by ELISA quantitation kits (Sun biomedical Technology Co., Ltd, Beijing, China) according to manufacturer instructions. The IgG level was measured in each case against a standard curve generated by employing known amounts of IgG. The protocols used to quantify IgG are similar to those described for cytokine detection.

### Detection of T cell subpopulation

For immunophenotyping, Peripheral Blood Mononuclear Cells (PBMCs) were washed in PBS supplemented with 0.1% sodium azide and 1% normal mouse serum, and incubated for 30 min at 4°C with one of the following monoclonal antibodies (mAbs) (all obtained from BD Pharmingen): fluorescein isothioncyanate (FITC)-conjugated mouse CD3^+^, PE-mouse CD4^+^, and PE-mouse CD8α. Cells were analyzed using a FACSCalibur flow cytometer (Becton Dickinson).

### Statistical analysis

Experimental data was expressed as mean ± S.D. Statistical analysis (one-way ANOVA) was performed using the GraphPad PRISM version 4.0 (GraphPad Software, San Diego, CA) to analyze the differences among the means of experimental groups. When a parameter of any of the experimental groups was significant, Dunnett's Multiple Comparison Test was used. Differences were considered to be significant at P < 0.05 and highly significant at P < 0.01.

## Abbreviations

sIgA: secretory immunoglobulin A; IgG: Immunoglobulin G; CFU: colony-forming unit; SPF: specific pathogen free; PBS: phosphate buffered saline; OPD: o-Phenylenediamine; PBMC: Peripheral Blood Mononuclear Cells

## Competing interests

The authors declare that they have no competing interests.

## Authors' contributions

TY, TS and QZ carried out the microbiological work and the animal studies. HZ, BM and TY conceived of the study. TY, QZ, and HZ designed the experiments. FC and TY performed the statistical analyses and prepared the figures. FC and TY wrote the draft of the manuscript. FC, JM and RD revised it for significant intellectual content. All authors read and approved the final version of the manuscript.
